# Enhancing intercultural competence of German medical students through innovative teaching on medical ethics with a focus on Muslim patients – a pilot study

**DOI:** 10.1186/s12910-024-01153-6

**Published:** 2024-12-23

**Authors:** Aysun Tekbaş, Arian Mauntel, Thomas Lehmann, Hans-Michael Tautenhahn, Utz Settmacher, Teresa Festl-Wietek, Anne Herrmann-Werner

**Affiliations:** 1 Department of General, Visceral and Vascular Surgery, Jena University Hospital, Friedrich Schiller University Jena, Am Klinikum 1, Jena, 07747 Germany; 2 Interdisciplinary Center of Clinical Research, Medical Faculty Jena, Research Programme “Clinician Scientist Programme”, Jena University Hospital, Friedrich Schiller University Jena, Salvador-Allende-Platz 29, Jena, 07747 Germany; 3 Institute for Medical Statistics, Computer and Data Sciences, Jena University Hospital, Friedrich Schiller University Jena, Bachstr. 18, Jena, 07743 Germany; 4 Department of Visceral, Transplantation, Thoracic and Vascular Surgery, University Hospital Leipzig, Liebigstr. 20, Leipzig, 04103 Germany; 5 TIME (Tübingen Institute for Medical Education), Medical Faculty Tuebingen, Eberhard Karls University Tuebingen, Hoppe-Seyler-Str. 3, Tuebingen, 72076 Germany; 6 Department of Internal Medicine VI/Psychosomatic Medicine and Psychotherapy, Tuebingen University Hospital, Eberhard Karls University Tuebingen, Osianderstreet 5, Tuebingen, 72076 Germany

**Keywords:** Intercultural competence, Muslim patients, Medical education

## Abstract

**Background:**

Effective healthcare delivery in today's diverse society necessitates healthcare providers' adeptness in navigating cultural and religious nuances in patient care. However, the integration of cultural competence training into medical education remains inadequate, particularly concerning the care of Muslim patients. In response, we introduce a novel educational intervention aimed at enhancing intercultural proficiency among medical students, emphasizing care for Muslim patients.

**Methods:**

The intervention comprised interactive seminars and simulated patient sessions. With a bespoke and the Cross-Cultural Competence of Healthcare Professionals (CCCHP-27) questionnaire the pre- and post-course intercultural competencies of *n* = 31 medical students of the Medical Faculty of Jena University were assessed. Additionally, there was a control group consisting of 34 students. Statistical analyses including descriptive statistics, paired samples t-tests, Wilcoxon tests, correlation analysis, Mann–Whitney U-tests, and multiple regression analysis were employed for data analysis.

**Results:**

Results of the bespoke questionnaire reveal significant improvements in intercultural knowledge (median pre 1.0 (0.6 – 1.6), median post 2.2 (2.4-2.8), *p* < 0.001) and in knowledge regarding Muslim patients (median pre 1.0 (0.5 – 1.5), median post 2.5 (2-3), *p* < 0.001) following the course. Regarding the CCCHP-27, students demonstrated a significant improvement in their skills, with pre-assessment score of 4.10 (± 0.47) and post-assessment score of 4.38 (± 0.40), *p* = 0.001. Female participants and those with limited prior experience demonstrated greater gains (*p* = 0.005 and *p* = 0.053). Notably, the incorporation of a session with a simulated patient garnered favorable feedback, affirming the efficacy of practical application in consolidating learning outcomes.

**Discussion:**

Our study emphasizes the importance of integrating intercultural competencies training into medical education and our findings underscore the efficacy of targeted educational interventions in enhancing intercultural competencies among medical students. For the assessment of intercultural competence, our bespoke questionnaire serves as a valuable addition to the German healthcare system.

**Conclusion:**

Implementation of similar interventions into medical curricula nationwide is imperative to address the needs of diverse patient populations effectively.

**Supplementary Information:**

The online version contains supplementary material available at 10.1186/s12910-024-01153-6.

## Introduction

The evolving dynamics of healthcare delivery within a pluralistic and transcultural society underscore the increasing significance of healthcare providers' interactions with immigrant patients. A 2021 study titled *"Muslimisches Leben in Deutschland 2020,"* conducted by the Federal Office for Migration and Refugees (BAMF), estimated that between 5.3 and 5.6 million Muslims lived in Germany, making up approximately 6.4 to 6.7% of the country’s total population [1]. Up to 30% of medical cases in certain healthcare settings involving individuals of the Islamic faith [2]. This demographic reality poses a mounting challenge for healthcare professionals who may find themselves navigating cultural and religious terrain unfamiliar to their training [3]. Especially Muslim patients have specific needs, that have to be considered. (Sunni) Islam emphasizes the importance of hygiene and upholding practices (such as daily prayer) that contribute to overall well-being. In medical contexts, Islamic principles highlight the necessity of safeguarding health while advocating for same-gender healthcare providers whenever possible, reflecting cultural and religious sensitivities [4–6]. Inadequate awareness of the cultural and religious nuances inherent in patient care not only exacerbates possible language barriers and communication hurdles but also engenders misconceptions regarding gender norms, notions of modesty, and ethical quandaries surrounding pivotal life stages [7]. The consequences of such gaps in understanding can reverberate through misinterpreted medical histories and flawed communication, culminating in potentially detrimental treatment outcomes [8, 9]. It may also result in disregarding the patient’s possible preferences. We have previously reported on this, using the example of potential conflicts arising for Muslim patients from the ingredients of medications due to special dietary habits (halal), along with a recommendation on how these issues could be addressed [5]. Additionally, patients often experience uncertainty and anxiety due to the unfamiliar processes in the medical environment. They frequently feel misunderstood [10]. On the other hand, improving the cultural competence of medical personnel can reduce disparities in the healthcare system [3].

A survey of 90 general practitioners regarding their subjective thoughts on the topic of "Muslim patients" with content analysis, revealed that many surveyed general practitioners perceive the treatment of Muslim patients as challenging [11]. They associate Muslim patients with linguistic communication barriers, different understandings of illness, and examination situations burdened by apprehension. Positive associations and unproblematic examination situations were noted less frequently. Consequently, the conclusion drawn is that Muslim patients are often depicted in a less reflective and frequently stereotypical manner due to cultural and religious contexts [11].

Addressing these challenges requires a concerted effort to enhance the cultural competence of healthcare practitioners, which also creates valuable learning opportunities for students. The documented effectiveness of cultural competence training in enhancing healthcare providers' cultural proficiency and mitigating health disparities is widely acknowledged [12]. Yet, the dearth of emphasis on this critical aspect of medical education within German universities leaves future physicians ill-equipped for the complexities of patient care in an increasingly diverse society [13]. Diverse approaches to addressing cultural competence and global health exist across German universities. While some focus on individual aspects separately, others offer integrated formats. Many universities provide optional courses driven by dedicated faculty and enthusiastic students, rather than structured integration into official curricula, which remains uncommon [14–16]. In 2017, the Hochschulrektorenkonferenz (HRK), the association representing German universities and higher education institutions, put forth recommendations aimed at enriching curricula through internationalization efforts. Specifically, they called for the deliberate integration of coursework centered on cultural competence and global health into medical education programs [17]. In their position paper, the “Gesellschaft für Medizinische Ausbildung” (GMA) Committee on Cultural Competence and Global Health addresses the growing significance of cultural and linguistic diversity in routine medical practice across Germany, Austria, and Switzerland [16]. The recommendations emphasize the integration of cultural competence and global health into medical education. This entails recognizing their synergies, embedding them into curricula, ensuring faculty qualification, adopting an interdisciplinary approach, and fostering research and evaluation. By linking these areas, medical students gain essential skills to navigate diverse patient populations and address global health challenges, contributing to comprehensive academic learning and patient-centered care [16].

To address these concerns, the introduction of the national competence-based catalogue of learning objectives for undergraduate medical education (NKLM) in 2015 represents a significant milestone [16, 18]. The NKLM has outlined competence-based learning objectives specifically tailored for undergraduate medical education in Germany, covering essential aspects of cultural competence and global health [16, 18]. This development marks a crucial step towards standardizing cultural competence education within medical curricula and ensuring that future healthcare professionals are equipped to navigate the complexities of providing care in diverse cultural contexts.

Within this context, the Cultural Competence Continuum offers a conceptual framework to describe the spectrum of cultural competence among individuals or organizations [19]. It typically presents cultural competence as a developmental process, where individuals or institutions progress from a state of cultural destructiveness (where differences are devalued and marginalized) to cultural proficiency (where differences are respected, valued, and integrated into practice) [19].

This continuum is particularly relevant when considering the care of Muslim patients and their families, as current studies show evidence of discrimination within the German healthcare system, affecting individuals of diverse backgrounds, including Muslims [20]. It emphasizes the importance of moving beyond mere tolerance of differences to genuinely understanding and integrating them into healthcare practices. For example, in the early stages of cultural competence, Muslim patients may encounter challenges if their cultural and religious needs—such as dietary restrictions, prayer practices, and gender preferences—are not considered. However, by incorporating intercultural competence into medical education, healthcare professionals can learn from the outset to better address these specific needs. This includes implementing practices that respect and accommodate religious observances, such as providing halal food and medication, respecting prayer times, and ensuring same-gender examinations, where possible [5].

It is also essential to evaluate intercultural competence through questionnaires, as they provide a systematic and standardized method for measuring participants' skills and attitudes towards potential intercultural teachings, thereby also enabling the assessment of the quality of possible interventions. Bernhard G. et al. were the pioneering group to develop a questionnaire aimed at assessing intercultural competencies within the German healthcare system (the CCCHP-27) [21]. While the majority of cultural competence models and instruments have originated in the United States, they inherently mirror the socio-cultural and political landscape of their development [21].

Grounded in the conceptual framework of cultural competence in healthcare, our study introduces a novel educational intervention. We hypothesize that the intervention will enhance medical students' overall intercultural competence, particularly in their interactions with Muslim patients.

By imparting essential knowledge and refining communication skills through real-world scenarios and interactions with a simulated patient (sp), our program endeavors to bridge the gap between theory and practice. Furthermore, our objective is to assess the effectiveness of our initiative in preparing the healthcare workforce to meet the diverse needs of contemporary patient care. For that, we conducted a thorough evaluation of students' pre- and post-course intercultural competencies using our own questionnaire, which probes participants' knowledge and skills in intercultural communication within the medical context, with a specific emphasis on interactions with Muslim patients. This aims to provide an additional means of assessing intercultural competence in the German healthcare system. Additionally, we utilized the validated CCCHP-27 [21] assessment tool to measure these competencies.

## Material and methods

### Study design (Fig. 1)

**Fig. 1:**
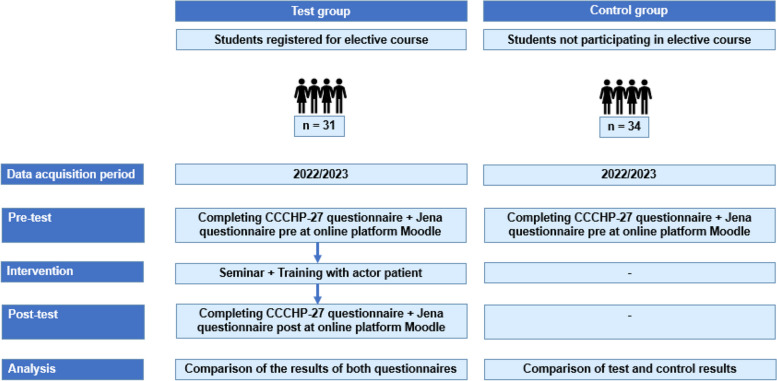
Study design

The course is offered at the Medical Faculty of Jena University Hospital as an elective seminar for students from the 6th semester onwards. It consists of an interactive seminar (timepoint 0) and a session with a simulated patient 1 week later (timepoint 1). Students completed the validated questionnaire (CCCHP-27) as well as the new, self-designed questionnaire (pre-test questionnaire). Subsequently, they participated in the seminar and the session with the simulated patient, after which they completed the post-test questionnaire and again the CCCHP-27.

For evaluation, the results of the pre- and post-questionnaires have been compared. Furthermore, a control cohort consisting of students from the 6th semester onwards was formed, comprising individuals who either did not intend to participate in the course or had not previously participated in it. They completed the pre-test questionnaire. Thus, the control group did not address the questions of the self-designed questionnaire regarding the simulated patient. The analyses conducted are exploratory in nature, serving as a longitudinal pilot study.

### Teaching concept (Appendix B)

The course, offered as an elective module at Jena University Hospital's surgical department, targets affective and cognitive aspects of knowledge, skills, and abilities in intercultural healthcare. Learning objectives include understanding ethico-religious aspects of patients in a hospital setting, recognizing specific behaviors related to these aspects, conducting medical procedures considering ethico-religious considerations, and demonstrating professionalism in patient treatment. It combines blended learning, interactive seminars, and simulated patient training with a maximum of 20 participants per session.

Prior to the course, participants engage in a *preparatory phase* on the Moodle® online learning platform, embracing blended learning techniques. During this 30-min session, they delve into independent study on Cholecystitis, exploring its intricacies in pathophysiology, diagnosis, and therapy, with a particular emphasis on surgical interventions. Additionally, they undertake the completion of the pre-test questionnaire and the CCCHP-27.

The seminar follows a sandwich approach [22], spanning 135 min and consists of an introductory phase, a main seminar with presentations and discussions, a midpoint survey, a deepening phase with case studies, and a concluding reflection phase.

A follow-up session involves simulated patient training, focusing on a Muslim patient with acute cholecystitis in an emergency department. The exercise helps participants treat a patient professionally while addressing ethico-religious aspects (such as gender preferences and language barriers) and, in doing so, understand the impact of cultural differences on communication and behavior, following the Cultural Competence Continuum.

In the session, up to 5 students are each designated for specific tasks:

- Group 1 conducts medical history-taking.
- Group 2 performs the necessary clinical examination and informs the patient.
- Group 3 explains the therapy to the patient.
- Group 4 provides information about the hospital treatment process.

Following time for gathering questions, the patient interaction forms the main part of the session and is concluded with feedback. The post-test questionnaire and again the CCCHP-27 are then filled out.

### Data collection

Data were collected from participants who willingly consented to participate. The participants were provided with clear instructions on how to complete the questionnaires, and their responses were anonymized to ensure confidentiality.

Thirty-one course participants completed both pre- and post-questionnaires, as well as the CCCHP-27 questionnaire. The control group consisted of thirty-four students, answering only to the pre-test questionnaire and the CCCHP-27.

### Assessment of intercultural competences

To assess the level of knowledge of students regarding intercultural competencies in general, as well as specifically concerning Muslim patients, we employ both a bespoke questionnaire developed internally and a rigorously validated multidimensional questionnaire (“Cross-Cultural Competence of Healthcare Professionals” (CCCHP-27) [21]).

### Development of own questionnaire

Relevant studies, articles, and existing tools related to intercultural competencies in the medical field, with a specific focus on interactions with Muslim patients, were examined [21, 23–25]. The information retrieved from the literature was used to design an own questionnaire for assessing intercultural competencies of medical students in Germany, as there is a scarcity of suitable tools.

The demographic data gathered comprised age, gender, academic level, assessment of previous experience, religious background, bilingual upbringing, and proficiency in additional languages. Moreover, the students’ motivation for participation in the course was assessed in the pre-test questionnaire (Table 1). The post-test questionnaire is identical to the pre-test questionnaire, with additional questions to evaluate the simulation.

**Table 1:** Items of own questionnaire to assess students' motivation before the course with response options and percentage (%) of given answers for *n* = 31 students of the test group (T) and *n* = 34 students of the control group (C) with respective *p*-value

Item No.	Sample Item	Options	Responses % T	Responses % C	*p*-value
M1	What importance do you personally attribute to intercultural communication?	Inconsequential	0	0	0.021
		Somewhat unimportant	0	8.8	
		Important	38.7	55.9	
		Very important	61.3	35.3	
M2	Do you think that an adequate amount of attention is devoted to the topic of medical ethics in medical school?	No, I have not yet come into contact with this topic as part of my studies	48.4	47.1	0.447
		Yes, the topic is addressed in the curriculum, but not enough	45.2	29.4	
		Yes, I have been sufficiently confronted with it during medical training	6.4	23.5	

In addition, questions were formulated to evaluate the general intercultural competencies of students, alongside their competencies tailored to interactions with Muslim patients, both pre- and post-course (Appendix A). The scale values ranged from 0 to 3, with 0 indicating insufficient knowledge and 3 representing very good knowledge.

### CCCHP-27 questionnaire [21]

The CCCHP-27 questionnaire has been validated as a reliable and comprehensive instrument for evaluating healthcare professionals' cultural competence [21]. Principal component analysis provided evidence for the construct validity of the CCCHP-27 questionnaire, revealing a comprehensive six-component structure comprised of 32 items, which collectively accounted for 50% of the total variance. The distinct facets of healthcare professionals' cultural competence encompass areas such as cross-cultural motivation/curiosity (CC-MC), attitudes (CC-A), skills (CC-S), emotions/empathy (CC-EE), and knowledge/awareness (CC-KA) as well as social desirability (SD). The scale values of each item range from 1 to 5, with high values indicating high cross-cultural competences [21].

The questionnaires’ components are as follows:

1. CC-MC: The items pertained to healthcare professionals' inclination towards offering culturally sensitive care, their interest in engaging with cross-cultural interactions, and their desire to enhance their comprehension when working with culturally diverse populations.
2. CC-A: This dimension proposed to reflect attitudes characterized by tolerance, appreciation, and respect for differences, as well as a favorable disposition towards embracing cultural diversity and other cultures.
3. CC-S: The CC-S was designed to assess healthcare professionals' ability to adapt to the cultural needs of their patients, their communication proficiency, and their capacity to allocate time for patient care.
4. CC-EE: The items centered around emotions and emotional responses related to diversity, encompassing feelings of comfort amidst challenges encountered in cross-cultural interactions and displaying multicultural empathy.
5. CC-KA: This component pertained to knowledge specific to culture and migration, an understanding of illness and health concepts, and awareness of one's own perceptions and values.
6. SD: The items reflect socially desirable content and was theorized to represent attitudes towards cross-cultural interactions.

### Data analysis

Statistical analysis was performed with SPSS software (IBM®, version 23, IBM Corporation, Armonk, NY, USA). Descriptive statistics were employed to summarize demographic information and frequencies. For continuous values, the paired samples t-test was used, with the mean and standard deviation reported (CCCHP-27). Due to the sample size, the t-test can be used without extensively checking for normality [26]. For ordinal variables, the median and interquartile range (IQR) were provided, and the Wilcoxon test was applied (own questionnaire). Correlation analysis was executed utilizing the Spearman test for assessing the relationships between 2 continuous or ordinal variables. Additionally, the Mann–Whitney-U-Test was employed to compare continuous or ordinal variables between 2 distinct groups. Multiple regression analysis was used to account for confounding variables. Significance was attributed to *p*-values < 0.05.

## Results

### Demographic data (Table 2)

**Table 2:** Demographic data of *n* = 31 study participants (test (T) group) and control (C) group (*n* = 34). M: male, f: female

	T group		C group	
Parameter	n	%	n	%
Gender				
m	8	25.8	11	32.4
f	23	74.2	23	67.6
Semester				
6th	18	58.1	16	47.1
7th	5	16.1	0	0
8th	2	6.5	6	17.6
9th	4	12.9	1	2.9
≥ 10th	2	6.4	11	32.4
Age				
20—24	24	77.4	27	79.4
25—29	5	16.1	5	14.7
≥ 30	2	6.5	2	5.9
Prior experiences				
Yes	21	67.7	27	79.4
No	10	32.3	7	20.6
Religious affiliation				
Islam	1	3.2	2	5.9
Other	30	96.8	32	94.1
Bilingual education				
Yes	4	12.9	3	8.8
No	27	87.1	31	91.2
Foreign languages				
None	2	6.5	0	0
1	11	35.5	17	50
2	10	32.2	9	26.5
≥ 3	8	25.8	8	23.5

In the test group (T), 31 participants were enrolled, consisting of 8 males and 23 females, while the control group (C) included 34 participants, with 11 males and 23 females. Participants in both groups represented various semesters, with a predominant presence in the 6th semester (T: 58.1%, C: 47.1%). Age distribution ranged from 20 to 33 years, with the majority falling within the 20 – 24-year-old bracket (T: 77.4%, C: 79.4%).

Moreover, a significant proportion of participants in both groups reported prior engagement in interactions with Muslim patients (T: 67.7%, C: 79.4%). The majority of participants did not identify with Islam (T: 96.8%, C: 94.1%). Although a minority grew up bilingual (T: 12.9%, C: 8.8%), a substantial majority demonstrated proficiency in more than one foreign language (T: 93.5%, C: 100%). Overall, both the test and control groups were comparable.

### Motivation

In the test group, 61.3% of the students considered the topic to be very important (question M1), while in the control group, this percentage was 35.3%. Furthermore, responses from the test group to question M2 indicated that the topic is not given enough attention in the medical curriculum (93.6%), compared to 76.5% in the control group. In total, these findings suggest a substantial level of interest in the subject matter (Table 1), which is not limited to Muslim students, as the majority of participants did not identify with the religion. Moreover, while a significant difference in motivation was observed between the control and test groups for M1 (*p* = 0.021), no such distinction was evident for M2 (*p* = 0.447).

### Efficiency of the course to enhance intercultural competence measured with own questionnaire

**Fig. 2:**
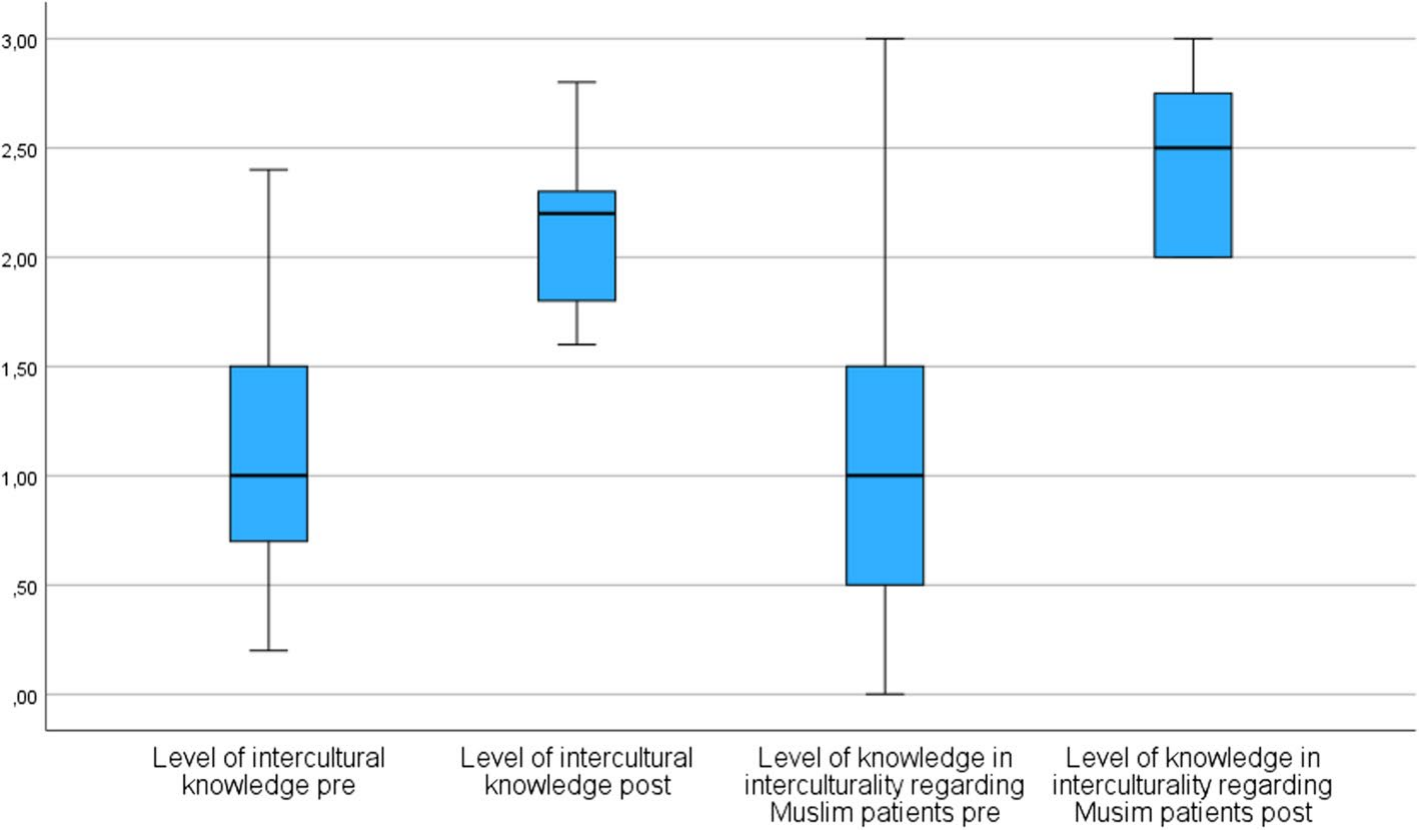
Gain in intercultural knowledge and knowledge regarding Muslim patients after the course in the test group, determined using the questions from the own questionnaires, *n* = 31

An enhancement in overall intercultural competence, both in general (median pre 1.0 (0.6 – 1.6), median post 2.2 (2.4 – 2.8)) and particularly concerning Muslim patients (median pre 1.0 (0.5 – 1.5), median post 2.5 (2 – 3)), was evident following the course (*p*-value < 0.001, Table 3, Fig. 2). However, the competencies of the control group were similar to those of the pre-test group (Tables 3 and 4).

**Table 3:** Median, interquartile range (25th – 75th percentile) and *p*-value of the total scores from the respective items of own pre- and post-test-questionnaires of *n* = 31 study participants. High values indicate high competences (range 0 – 3)

Dimension	Median (25th-75th percentile) pre	Median (25th-75th percentile) post	*p*-value
Level of intercultural knowledge	1.0 (0.6 – 1.6)	2.2 (2.4 – 2.8)	< 0.001
Level of knowledge in interculturality regarding Muslim patients	1.0 (0.5 – 1.5)	2.5 (2—3)	< 0.001

**Table 4:** Median and interquartile range (25th – 75th percentile) of the total scores from the respective items of own questionnaire of control group (C, *n* = 34) and post-test group (*n* = 31) with *p*-values. High values indicate high competences (range 0 – 3)

Dimension	Median (25th-75th percentile) C	Median (25th-75th percentile) post	*p*-value
Level of intercultural knowledge	1.1 (0.6 – 1.6)	2.2 (2.4 – 2.8)	< 0.001
Level of knowledge in interculturality regarding Muslim patients	1.0 (0.5 – 1.5)	2.5 (2—3)	< 0.001

To evaluate the enhancement of overall intercultural competencies (sum of IK1-IK5 and IM1-IM2) in relation to age, gender, prior experience, and bi-/multilingualism, the change in competence was calculated as the difference between post and pre-assessment scores. Notably, no significant correlation was detected (*p* = 0.144) between the augmentation of overall intercultural competencies and age, with the correlation coefficient indicating a slight negative association (*r* = -0.269), suggesting that younger students tend to demonstrate higher intercultural competences than older ones. Female participants exhibited a more pronounced benefit from the course in terms of increased intercultural competence (*p* = 0.005). An intriguing observation was that students with limited prior experience showed greater gains from the course (*p* = 0.053). The absence of a significant relationship between bilingualism (*p* = 0.237) and the enhancement of intercultural competence is also notable. Similarly, the proficiency in foreign languages and the benefits derived from the course exhibit no discernible correlation (*p* = 0.241). Furthermore, it's worth highlighting that the correlation coefficient indicates a negative trend (*r* = -0.217), suggesting that individuals speaking more languages tend to experience a lesser increase in intercultural competence.

### Efficiency of the course to enhance intercultural competence measured with CCCHP-27 questionnaire

In relation to the dimensions encompassing attitude, emotions, knowledge, and social desirability as measured by the CCCHP-27 questionnaire, no notable disparities were observed between the pre- and post-survey evaluations conducted with the students. While there was a minor disparity in the knowledge dimension, with unexpectedly slightly higher scores recorded in the pre-test (4.4 ± 0.5) compared to the post-test (4.2 ± 0.5), this difference did not reach statistical significance (*p* = 0.223, Table 5). Given the consistently high score values across both tests, it is more apt to characterize the findings as indicative of stability. Nonetheless, a noteworthy increase in skills was evident (*p* = 0.001). Furthermore, the augmentation in motivation, though marginally above the significance threshold, suggests a discernible overall improvement (mean pre 4.4 ± 0.5, post 4.5 ± 0.5, *p* = 0.057).

**Table 5:** Mean, standard deviation (SD) and *p*-value of the total scores from the respective items of the dimensions of the CCCHP-27 questionnaire pre and post of *n* = 31 study participants. Higher values indicate enhanced cross-cultural competence (range 1 – 5)

Dimension	Mean (± SD) pre	Mean (± SD) post	*p*-value
Motivation	4.4 (± 0.5)	4.5 (± 0.5)	0.057
Attitude	3.5 (± 0.8)	3.6 (± 0.8)	0.184
Skills	4.1 (± 0.5)	4.4 (0.4)	0.001
Emotions	3.6 (± 0.5)	3.8 (± 0.4)	0.094
Knowledge	4.4 (± 0.5)	4.2 (± 0.5)	0.223
Social desirability	4.4 (± 0.5)	4.5 (± 0.4)	0.298

The comparison of the scores of the dimensions from the post-survey questionnaires with the control group, on the other hand, yielded significant values except for the dimension "attitudes" (Table 6). The main difference between the test and control groups regarding the dimensions of motivation, attitude, skills, emotions, and social desirability of the CCCHP-27 lies primarily in motivation, as measured by question M1, where the test group demonstrates higher motivation (Table 7). For the dimension of knowledge, there is no significant dependency on motivation evident (*p* = 0.660), with the means in both groups already quite high (T: 4.4 ± 0.5, C: 4.5 ± 0.5).

**Table 6:** Mean, standard deviation (SD) and *p*-value of the total scores from the respective items of the dimensions of the CCCHP-27 questionnaire of *n* = 31 study participants after the course (post) and control group (C, *n* = 34). Higher values indicate enhanced cross-cultural competence (range 1 – 5)

Dimension	Mean (± SD) C	Mean (± SD) post	*p*-value
Motivation	4 (± 0.8)	4.5 (± 0.5)	0.003
Attitude	3.4 (± 0.9)	3.6 (± 0.8)	0.357
Skills	3.5 (± 0.9)	4.4 (0.4)	< 0.001
Emotions	3.5 (± 0.8)	3.8 (± 0.4)	0.039
Knowledge	4.5 (± 0.5)	4.2 (± 0.5)	0.050
Social desirability	4 (± 0.5)	4.5 (± 0.4)	< 0.001

**Table 7:** Regression analysis to assess dependency of differences in the dimensions of the CCCHP-27 on m1 and m2

Dependent variable	Constant	*p*-value	95% Confidence interval
Motivation	M1	< 0.001	0.295 – 0.856
	M2	0.487	-0.275 – 0.132
Attitude	M1	0.006	0.151 – 0.844
	M2	0.104	-0.488 – 0.046
Skills	M1	0.017	0.078 – 0.756
	M2	0.059	-0.010 – 0.512
Emotions	M1	0.013	0.079 – 0.646
	M2	0.559	-0.282 – 0.154
Knowledge	M1	0.660	-0.178 – 0.280
	M2	0.080	-0.333 – 0.020
Social desirability	M1	0.044	0.006 – 0.456
	M2	0.167	-0.052 – 0.295

### Evaluation of the training with the simulated patient

The assessment of the instructional session with the simulated patient was conducted via the post-test questionnaire, wherein elevated numerical scores (ranging from 0 to 3) denoted favorable responses. The prevailing sentiment among students indicated a meaningful application of acquired skills with the simulated patient, reflected by a median score of 3 (IQR: 2–3, 25th–75th percentile). Moreover, the utilization of a simulated patient demonstrably elevated the efficacy of learning outcomes, with a median score of 3 (IQR: 2–3, 25th–75th percentile), fostering a profound comprehension of the subject matter, as indicated by a median score of 2 (IQR: 2–3, 25th–75th percentile). Students lauded the well-constructed nature of the case, yielding a median score of 2 (IQR: 2–3, 25th–75th percentile), and deemed it conducive to comprehensive engagement with the material, garnering a median score of 2 (IQR: 2–3, 25th–75th percentile).

## Discussion

Intercultural competence is undeniably crucial for effectively treating our diverse patient population [27]. Our study presents a comprehensive educational intervention aimed at enhancing German medical students' intercultural competence, particularly in their interactions with Muslim patients. The intervention encompasses a blended learning approach, interactive seminars, and simulated patient training, with a focus on bridging theory and practice. The results of the study demonstrate notable improvements in overall intercultural competence among participants, as evidenced by objective measures using questionnaires. The acquired knowledge was successfully applied in practice.

The study's findings also shed light on the differential impact of the intervention based on demographic factors such as age, gender, and prior experience. The observed greater improvement in intercultural competence among younger students and female participants highlights the need for tailored educational approaches that take into account individual differences and learning preferences. Younger individuals often exhibit higher levels of openness to new experiences and adaptability, which are critical traits for developing intercultural competence. The increasing globalization and internationalization of higher education make cross-cultural interactions inevitable for young people. Developing intercultural competence is essential for students to engage with international peers, adapt to diverse academic settings, and prepare for multicultural workplaces in the future. Programs like study abroad or international exchanges are crucial in fostering these skills by providing direct exposure to diverse cultures, enhancing adaptability and global awareness [28, 29]. A study examining the gains in intercultural competence through studying abroad found that being female was a significant positive predictor of intercultural competence development. Female participants were more likely to demonstrate greater improvements compared to their male counterparts, potentially due to higher engagement with program elements such as language immersion and mentoring activities [30]. The lack of significant correlation between bilingualism or proficiency in foreign languages and the enhancement of intercultural competence suggests that language proficiency alone may not be sufficient to navigate the complexities of cross-cultural communication in healthcare settings. However, there is extensive literature on the cognitive and linguistic benefits of bilingualism in everyday life [31]. Language proficiency is essential for developing intercultural competence, enhancing communication and challenging perceptions, expressions, and interactions in both native and second languages. It promotes the use of alternative communication strategies and encourages a more flexible worldview. Language learning helps break habitual thinking, fostering a deeper understanding of different cultures [32].

An intriguing observation was that students with limited prior experience showed greater gains from the course (*p* = 0.053), likely because they begin at a lower baseline and experienced a steeper learning curve during the course.

The positive feedback received for the simulated patient training session indicates its value in providing students with practical opportunities to apply their newly acquired skills in a realistic clinical context [33]. This experiential learning approach fosters a deeper understanding of cultural diversity and its impact on patient care, preparing students to real-world clinical encounters with confidence and sensitivity.

Research indicates that training programs focusing on intercultural competence for healthcare professionals can lead to reduced stress and anxiety levels, benefiting both patients and providers alike [34]. Nonetheless, integrating cultural competence teaching into medical school curricula poses significant challenges, including limited resources, time constraints, and competing educational priorities. To address these hurdles, there's a pressing need for enhanced sharing of experiences and best practices among European medical schools [13]. Germany, in particular, has yet to address these challenges adequately, lacking a unified communication curriculum within its medical education sector [11]. Furthermore, there remains a scarcity of German research groups within the medical sphere that specifically address this thematic concern.

Fischer et al. illustrated that comprehensive awareness of individual, regional, cultural, and denominational disparities, coupled with a nuanced approach toward patients and their families, along with collaborative engagement with hospital chaplains, holds the potential to substantially enrich the doctor-patient rapport [35]. Such considerations warrant diligent attention, particularly when navigating interactions with individuals of diverse religious affiliations within the context of Germany's secularized society [35]. In the Bachelor's thesis by Klatzer, it is highlighted that delivering transcultural care goes beyond mere training [36]. It necessitates a holistic institutional dedication to openness and transformation across all levels. This involves actively engaging migrants in expressing their preferences for therapy and personal care. Additionally, both staff members and organizational standards must embrace a transcultural orientation to facilitate meaningful change. The overarching objective is not to establish uniform models for transcultural care but rather to seamlessly integrate this concept into existing care frameworks [36].

Ultimately, the literature underscores a pressing necessity to incorporate interculturality education into the curriculum for German medical students. While theoretical discussions regarding the integration of interculturality in medicine exist, the practical application remains scarce, limited, and primarily undertaken at an individual level within academic institutions.

This gap indicates a critical need for systematic efforts to address intercultural competence within medical education. There is an absence of widespread interest among faculties across Germany in prioritizing this aspect of medical training. Additionally, the development of comprehensive guidelines outlining the pertinent topics to be covered in interculturality education is lacking. Without concerted efforts to bridge this divide between theory and practice and to institutionalize intercultural competence training within medical curricula, healthcare professionals may struggle to effectively navigate the diverse cultural landscapes encountered in clinical practice. Consequently, addressing these deficiencies is imperative to ensure the provision of equitable and culturally sensitive healthcare services to an increasingly diverse patient population. The NKLM might offer an opportunity for Germany in this regard.

Our course, as is common in the landscape of medical education in Germany, remains optional. However, with our teaching concept and study, we are precisely addressing these aspects and laying the groundwork for further exploration of these topics. Furthermore, the results presented herein furnish a robust foundation for its integration into the national curriculum. Nonetheless, expanding the course further to encompass additional cultures and religions is imperative for comprehensive inclusivity and effectiveness.

While our teaching concept and the evaluation based on (bespoke) questionnaires represent a milestone in the implementation of interculturality in medical education in Germany, there are limitations in the design of our study. For instance, our bespoke questionnaire is not validated yet. Furthermore, the relatively small sample size of 31 students in the test group may limit the generalizability of our findings. While a larger number of students participated in the course, not all opted to take part in the study, potentially introducing bias. The absence of randomization in the control group raises considerations regarding the comparability of the two groups, particularly concerning motivation levels. It is conceivable that individuals in the test group, owing to their heightened interest and familiarity with the topic, may have been more inclined to participate in the elective course, potentially contributing to their superior performance in the CCCHP-27. However, overall, the high motivation underscores the importance of integrating cultural competence training more comprehensively into German medical curricula to meet the growing demands of a diverse patient population.

Future research with larger, more diverse samples and randomized control groups, as well as validated questionnaires, could further elucidate the effectiveness of such interventions and help refine educational approaches in this critical area.

## Conclusion

The new teaching concept enhances the intercultural competencies, particularly in terms of the skills, of medical students. The topic holds high relevance in our diverse society. Therefore, it is necessary to implement such teaching concepts into medical curricula nationwide, rather than keeping them solely as elective courses, as is currently the case.

## Supplementary Information

Supplementary Material 1.

## Data Availability

The datasets analysed during the current study are available on request from the corresponding author.
