# Supplementary material for: Enhancing intercultural competence of German medical students through innovative teaching on medical ethics with a focus on Muslim patients – a pilot study
Source: BMC Med Ethics. 2024 Dec 23;25:152. doi: 10.1186/s12910-024-01153-6 (PMC11664812; doi:10.1186/s12910-024-01153-6)
Supplement: Supplementary file 1 — Supplementary Material 1. [file 12910_2024_1153_MOESM1_ESM.docx]

Appendix A: Items of own questionnaire to assess students’ level of intercultural knowledge and in interculturality regarding Muslim patients before and after the course.

| Component | Item No | Sample Item |
| --- | --- | --- |
| Level of intercultural knowledge | IK1 | How do you assess your ability in intercultural communication? |
|  | IK2 | How do you assess your level of knowledge regarding conflict resolution strategies in the context of misunderstandings in intercultural settings with patients? |
|  | IK3 | How confident do you feel in conducting a culturally sensitive physical examination? |
|  | IK4 | How confident do you feel in dealing with culturally sensitive patient information and counseling? |
|  | IK5 | How competent do you feel in working with interpreters in the medical context? |
| Level of knowledge in interculturality regarding Muslim patients | IM1 | How do you assess your level of knowledge regarding sensitization to culturally specific aspects of Muslim patients? |


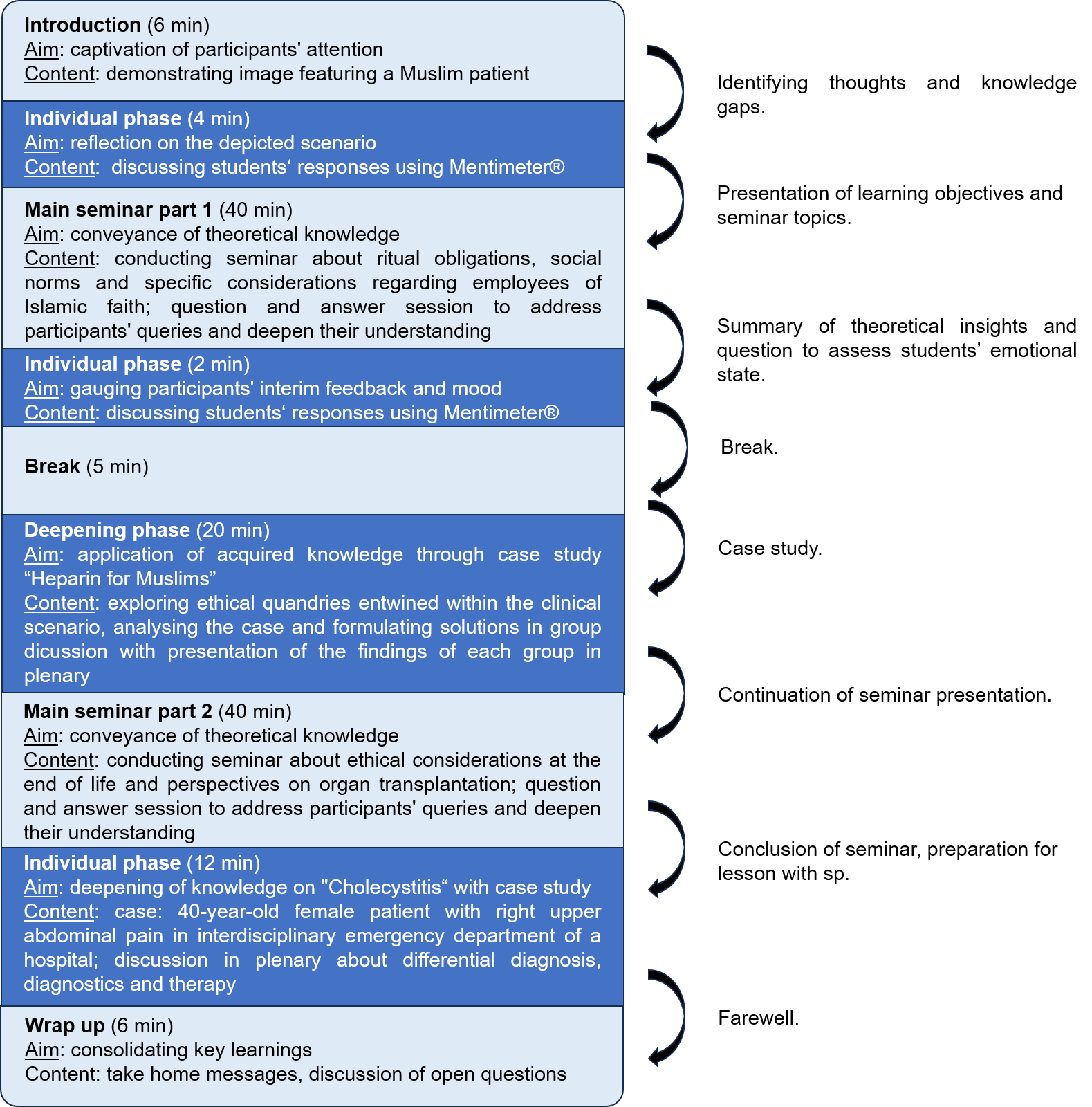


Appendix B: Teaching concept as sandwich approach [16]. Mentimeter®: interactive presentation and polling tool. Case “Heparin for Muslims”[24]: administration of porcine-derived heparin to a Muslim patient, potentially conflicting with Islamic beliefs when alternatives are available. Sp: simulated patient.
